# HIV-related perceived stigma and associated factors among patients with HIV, Dilla, Ethiopia: A cross-sectional study

**DOI:** 10.1016/j.amsu.2021.102921

**Published:** 2021-10-08

**Authors:** Yigrem Ali Chekole, Desalegn Tarekegn

**Affiliations:** a Department of Psychiatry, College of Health Science and Medicine, Dilla University, Dilla, Ethiopia; b Department of Midwifery, College of Health Science and Medicine, Dilla University, Dilla, Ethiopia

**Keywords:** Ethiopia, Perceived stigma, HIV/AIDS, AIDS, Acquired Immune Deficiency Syndrome, AOR, Adjusted Odds Ratio, ART, Anti-retroviral Therapy, CI, Confidence Interval, COR, Crude Odds Ratio, HIV, Human Immune Viruses, PLWH, People Living With HIV, SPSS, Statistical Package for the Social Sciences, WHO, World Health Organization

## Abstract

**Introduction:**

Understanding HIV-related perceived stigma has importance in improving the quality of patients and provides a better tackling of HIV stigma. Therefore; the study aimed to assess the prevalence and associated factors of perceived stigma among Patients with HIV attending the clinic at Dilla University Referral Hospital in Ethiopia 2019.

**Method:**

In this Institution based cross-sectional study, a 10-item perceived HIV stigma scale was used to assess HIV-related perceived stigma. Oslo social support scale was used to assess social support related factors. Bivariate and multivariate binary logistic analysis was done to identify associated factors to HIV-related perceived stigma.

**Results:**

The prevalence of HIV-related perceived stigma by using perceived HIV stigma scale among patients with living HIV was 42.7%. Patients who are age groups 25–30 years (AOR = 2.8, 95% CI: 5.72–11.5), age groups 31–39 years (AOR = 1.11, 95% CI: 1.26,4.65), Females (AOR = 2.4, 95% CI: 1.28–4.33), divorced marital status (AOR = 8.9, 95% CI: 3.52–10.61), widowed marital status (AOR = 3.0, 95% CI: 2.74–7.60), Primary educational status (AOR = 7.5,95% CI: 3.45–9.74) and Study participants those who use alcohol (AOR = 1.0 95% CI: 1.57–2.11) were more likely to have HIV-related perceived stigma.

**Conclusion:**

This calls a holistic approach to the prevention and intervention of HIV-related perceived stigma. Emphasis should also be given for HIV-related perceived stigma.

**Registration:**

This study was registered research registry with the registration number (researchregistry7112).

## Highlights

• The prevalence of HIV-related perceived stigma was very high.
• Age groups 25–30 years were more likely to have perceived stigma.
• Females were more likely to have perceived stigma as compared to males.
• Divorced participants with marital status were more likely to have perceived stigma .

## 1 Introduction

HIV-related perceived stigma remains pervasive and affects people with HIV the right to fully participate in their communities, affecting all aspects of people's lives, including access to treatment and care, and access to work Negative impact of HIV-related stigma [1] poor HIV outcome [2]. People living with HIV may feel shame and fear of discrimination [3].

HIV-related perceived stigma my lead to a series of consequences such as non-disclosure of HIV infection seclusion, depressive symptoms, and suicidal ideation and attempt [4]. Due to this effect, PLWH has to cope both with the manifestations of the disease, complex treatment regimen and societal stigma at the same time [[5], [6]]. HIV/AIDS-related stigma and discrimination can be directed at infected people as well as their friends, families, caretakers and others [[7], [8]]. It greatly affects the quality of life of them, their family members and the health care providers who work with them [9] and cause serious care limitation [10]. Stigma can also cause serious social and psychological damage and significantly increases loneliness, depression, anxiety, non-disclosure of HIV status and overall poor health outcomes [[11], [12]].

Sub Saharan Africa contributed 76% (29 million) of the total HIV infected people [13]. In Ethiopia, the adult prevalence rate is estimated at 2.4% and the incidence rate is 0.29% [14].

A recent systematic review found that over the last decade, evidence-based effective programming to reduce stigmatizing and discrimination attitude has expanded substantially [15]. However, almost no country has prioritized activities to reduce or eliminate them in their national plans or program [16]. People who have stigma report a range of negative effects including loss of income or job, Isolation from communities and inability to participate as a productive member of society [17].

Globally 30%–80% of them know-how stigma during their lifetime [18]. A study undertaken among North Bengal medical college attending ART centre revealed that 25.8% had perceived stigma [19]. The study in Iran among women living with HIV reveals 69.7% [20].

A study in Chennai substantiated the perceived stigma was 26% of them had adept stigma [21]. Other studies reveal the prevalence of perceived stigma among people living with HIV attending ART clinic at the University of Port Harcourt Teaching Hospital, Nigeria is 59.9% [22]. A quantitative descriptive and cross-sectional study in Ethiopia, Addis Ababa were non-adherent and adherent to ART medication 36.2% and 10% perceived stigma respectively [23]. Today, evidence on prevalence and associated factors HIV-related perceived stigma among patients with HIV attending ART clinic is still in demand. Therefore; the study aimed to assess the prevalence and associated factors of perceived stigma among patients with HIV attending the clinic at Dilla University Referral Hospital.

## 2 Methods

### 2.1 Protocol and registration

The study was conducted based on the Strengthening the Reporting of Cohort Studies in Surgery (STROCSS 2019 Guideline) protocols [24]. This study was registered research registry with the registration number (researchregistry7112).

### 2.2 Study design and setup

An institutional-based cross-sectional study was conducted at Dilla University Referral Hospital Anti-retroviral clinic from April–May 2019. Dilla University Referral Hospital is found in Dilla Town (the capital of Gedeo Zone) Southern National's Nationalities and People Region and away 360 km from Addis Ababa, the capital city of Ethiopia. Patients receiving inpatient treatment and critically ill patients with the difficulty of communication were excluded.

### 2.3 Sample size determination and sampling technique

It was determined by Level of significance (0.05), Power (0.50) with z = 95% confidence interval and the value of ‘’p’’ (p = proportion of prevalence) was taken as the prevalence of perceived stigma among People Living with HIV which was found to be 61.1% (done in Jimma town, Ethiopia) [25]. Then by adding 10% of non-respondents then, the total sample size for this study was 403. The study also used a systematic random sampling technique to select study subjects.

### 2.4 Data collection and instruments

A semi-structured questionnaire was used to assess HIV-related perceived stigma felt by HIV patients. The instruments had included socio-demographic characteristic which mainly focuses on age, sex, education, occupation, marital status, religious view of the study participants, and others. Oslo item 3 social support scales which is the 3-item questionnaire and HIV stigma index validation survey and 10-item perceived HIV stigma scale to measure the outcome variable were used.

The outcome variable, HIV-related perceived stigma felt by HIV patients, was collected by 10-item perceived HIV stigma scale that consisted of four-point Likert scale questions (1 = strongly disagree, 2 = disagree, 3 = agree 4 = strongly agree) of their HIV status. The Cronbach alphas of 10-item perceived scale were ranged from 0.86 to 0.94 which was validated in different setting, languages, and population [[26], [27]].

Social support was collected by Oslo-3 item social support scale, it is 3 item questionnaires, commonly used to assess social support and it has been used in several studies, the sum score scale ranging from 3 to 14, which has 3 categories: poor support 3–8, moderate support 9–11 and strong support 12–14 [28]. Internal consistency (Cronbach alpha) of Oslo-3 in the current study is 0.85.

### 2.5 Data quality assurance

The pretest was done on 5% of the sample size. The training was given to the data collectors and supervisors on the data collection tool and sampling techniques. Supervision was held regularly during the data collection period by the researcher. The data were cross-checked for completeness and consistency daily.

### 2.6 Statistical analysis and processing

The coded data were checked, cleaned by entering into epi.info version 7.1 and then exported into Statistical Package for the Social Sciences (SPSS window version 20).

Descriptive statistics were used to summarize tables and figures and statistical summary measures were used for presentation. Association of HIV-related perceived stigma variables and demographic characteristics were analyzed using chi-square, fisher's exact test, and binary logistic regression with odds ratio and 95% CI in the univariate analysis.

Multivariate logistic regression analysis was carried out to examine the associations between each independent variable and the outcome variable. The model was checked for fitness with R-squared value was an R-squared value greater than 50% considered as good. Hosmer and Lemshow goodness of fit test was also used to check the model fitness. All variables with a p-value of ≤ 0.25 in the bivariable analysis were considered as the candidate for multivariable regression to control possible confounders. Finally, variables with a p-value of <0.05 were as having a statistically significant association with HIV-related perceived stigma at corresponding 95% CI.

### 2.7 Ethical clearance

Ethical approval was obtained from the Institutional Review Board of Dilla University and Referral Hospital. The purpose and importance of the study were explained to each participant before they proceed into actual activities. Confidentiality was maintained by anonymous questionnaire and informed consent was obtained from each participant.

## 3 Results

A total of 403 participants were interviewed and responded for questionnaires with response rate was 100%.

### 3.1 Socio-demographic characteristics

Most of the study subjects 206 (51.1%) participants were females. 135 (33.5%) respondents were at the age of >39 years, 206 (51.1%) were married and 193 (47.9%) respondents were orthodox in religion. Concerning ethnicity, 193 (47.6%) and 112 (27.8%) of them were from Oromo and Gedeo ethnic group, respectively. The majority 182 (45.2%) respondents had secondary school education, 124 (30.8%) participants were a government employee. Majority of the total respondents 161 (40.0%) of them were living with their children 159 (39.5%) were have poor social support and 244 (60.5%) were have strong social support more than half of the respondents 223 (55.3%) were use substance and 192 (47.6%) were the second stage of HIV (Table 1**)**.

**Table 1:** Socio-Demographic characteristics of study participant in ART clinic 2019.

Variable		Frequency	Percentage (%)
Age	18–24	30	8.7
	25–30	124	30.8
	31–38	130	32.3
	>39	135	33.5
Sex	Male	197	48.9
	Female	206	51.1
Marital status	Single	102	25.3
	Married	206	51.1
	Divorced	56	13.9
	Widowed	39	9.7
Religion	Orthodox	182	45.2
	Protestant	123	30.2
	Muslim	90	23.3
	Catholic	8	2.0
Ethnicity	Gedeo	133	33.0
	Oromo	150	37.2
	Amara	85	21.1
	Other^A^	14	3.5
Educational status	Can't read and write	8	2.0
	Primary education	34	8.4
	Secondary education	182	45.2
	Higher education	179	44.4
Occupation	Un employee	58	14.4
	Governmental employee	130	32.3
	Retire	22	5.5
	Business man	103	25.6
	Student	34	8.4
	House wife	47	11.7
	Other^B^	9	2.2
Income	>1000	73	18.1
	1000–2500	87	21.6
	2500–4000	115	28.5
	<4000	128	31.8
Living status	With family	102	25.3
	Alone	117	28.8
	With relative	26	5.7
	With children	162	40.2

Other^A^ = Silte, Tigre, Gurage, Other^B^ = farmer, daily labor**,** ART = Ant-retroviral Therapy, DURH = Dilla University Referral Hospital.

### 3.2 Prevalence of HIV-related perceived stigma among people living with HIV

**Fig. 1:**
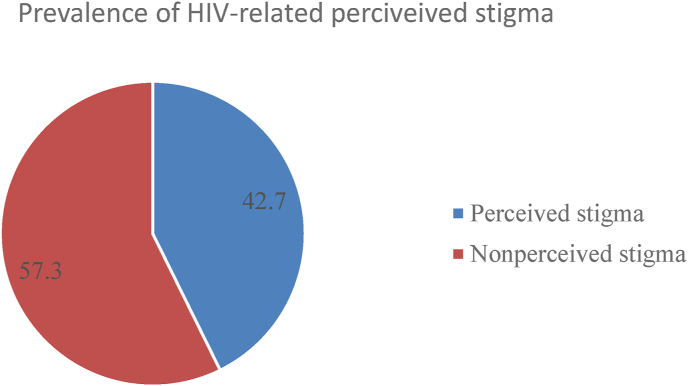
Prevalence of HIV-related perceived stigma among People living HIV attending ART clinic 2019.

The overall prevalence of Perceived Stigma was found to be 169 (42.7%) (Fig. 1).

### 3.3 Factors associated with HIV-related perceived stigma among people living with HIV

In Bivariate analyses, age, sex, marital status, occupation status, ethnicity, educational status, income, living status, HIV stage, substance use, social support and living status were analyzed. Multivariate logistic regression was also used to analyze associations between variables which have a p-value of <0.2 in Bivariate logistic regression. After adjusting for possible covariates, age, sex, marital status, ethnicity, educational status, occupational, living status, HIV stage was significantly associated with HIV-related perceived stigma among patients living with HIV with p-value<0.05.

Age groups 25–30 years were 2.8 times more likely to have perceived stigma as compared to age 18–24. (AOR = 2.8, 95% CI: 5.72–11.5).

Age groups 31–39 years were 1.1 times more likely to have perceived stigma as compared to age 18–24. (AOR = 1.11, 95% CI: 1.26,4.65).

Females were 2.36 times more likely to have perceived stigma as compared to males (AOR = 2.36, 95% CI: 1.28–4.33).

Study participants those had divorced marital status were 8.93 times more likely to have perceived stigma as compared to a single (AOR = 8.93, 95% CI: 3.52–10.61).

Study participants those had widowed marital status were 2.99 times more likely to have perceived stigma as compared to a single (AOR = 2.99, 95% CI: 2.74–7.60).

Primary educational status 7.5 times more likely to develop perceived stigma as compared to participants who can't read and write (AOR = 7.5, 95% CI: 3.45–9.74).

Study participants those who use alcohol were 1.01 times more likely to have perceived stigma as compared to khat (AOR = 1.01 95% CI: 1.57–2.11) (Table 2**)**.

**Table 2:** Bivariate and Multivariate analysis of factors associated with HIV-related Perceived Stigma among People living HIV attending ART clinic 2019.

Variables		Perceived stigma		COR (95%CI)	AOR (95%CI)
		No	Yes		
Age (n = 403)	18–24	11 (5%)	3 (2%)	1	1
	25–30	61 (26%)	63 (37%)	3.78 (1.0–4.23)	2.88 (5.72–11.5)**
	31–39	86 (37%)	44 (26%)	1.87 (0.50–7.07)	1.11 (1.26–4.65)**
	>39	73 (32%)	62 (36)	3.11 (0.83–11.66)	1.97 (0.457–8.54)
Sex (n = 403)	Male	115 (50%)	82 (48%)	1	1
	Female	116 (50%)	90 (52%)	1.08 (0.73–1.61)	2.36 (1.28–4.33)**
Marital status (n = 403)	Single	68 (29%)	34 (20%)	1	1
	Married	124 (54%)	82 (48%)	1.32 (0.80–2.18)	1.55 (0.74–2.54)
	Divorced	19 (8%)	37 (22%)	3.90 (1.95–7.76)	8.93 (3.52–10.61)***
	Widowed	20 (9%)	19 (8%)	1.90 (0.9–4.03)	2.99 (2.74–7.60)**
Educational status (n = 403)	Can't read and write	4 (2%)	4 (2)%	1	1
	Primary education	8 (3%)	26 (11%)	3.25 (0.66–16.04)	7.50 (3.45–9.74)***
	Secondary education	114 (49%)	68 (40%)	0.60 (0.14–2.46)	6.11 (0.409–9.258)
	Higher education	105 (45%)	74 (43%)	0.71 (0.17–2.91)	8.39 (0.54–12.33)
Substance use (n = 403)	Khat	56 (24%)	66 (38%)	1	1
	Alcohol	70 (30%)	79 (46%)	1.61 (1.04–2.50)	1.01 (1.57–2.11)*
	Cigarette	105 (45%)	27 (16%)	0.54 (0.30–0.96)	0.18 (0.07–1.46)
HIV stage (n = 403)	Stage 1	104 (45%)	92 (53%)	1	1
	Stage 2	93 (40%)	76 (44%)	0.92 (0.61–1.4)	1.48 (0.870–2.53)
	Stage 3	34 (15%)	4 (2%)	0.13 (0.05–0.39)	0.14 (0.041–1.51)

P*<0.05, P**<0.01, P***<0.001, HIV=Human Immune Virus.

## 4 Discussion

The study has tried to determine the prevalence of HIV-related perceived stigma and associated factors among people living with HIV attending Anti-retroviral clinic at Dilla University Referral Hospital.thus the prevalence of HIV-related perceived stigma was found to be 42.7%.

According to this study, the prevalence of HIV-related perceived stigma and associated factors among people living with HIV attending Anti-retroviral clinic was lower than the study conducted in Jimma Town was 61.1% [25] and in Nigeria 59.9% [22]. The difference might be due to the socio-economical status of the study setting, the sample size of the study, the influence of cultural and religious norms of the society.

The study is higher than A study conducted in Addis Ababa (36.2%) and 10% [23] The reason for the noted difference might be the attitude of the society in the study area, cultural variation, and educational status of the society and norms of the society.

Age groups 25–31 and 31–39 years were 2.8 and 1.1 times more likely to have HIV-related perceived stigma as compared to age 18–24 years respectively [25]. The reason noted that this level of age is high productive level; in terms of work, family and social relationship within the society accordingly and expect more role at this level of age.

Females were 2.4 times more likely to have HIV-related perceived stigma as compared to males [20]. The hormonal difference which may play important and are more vulnerable for gender discrimination, and neglect. They may suffer more perceived stigmas because the community views them as having been promiscuous at least once in their life when they are infected with HIV.

Study participants those had divorced marital status were 8.9 times more likely to have HIV-related perceived stigma as compared to singles [20]. This might be due to infected with this HIV might cause divorcing of study participants. Study participants those had widowed marital status were 3.0 times more likely to have perceived stigma as compared to singles [20]. This is might be due to decreasing of family and friend support with being HIV infected and widowed due to its morbidity and mortality.

Primary educational status was 7.5 times more likely to develop HIV-related perceived stigma as compared to participants who can't read and write the study participants [20].it might be little awareness of having HIV makes them stigmatize, cultural influence, the norm of the society and educational status.

Study participants those who use alcohol were 1.0 times more likely to have HIV-related perceived stigma as compare to khat [20]. This might be that drinking alcohol and khat chewing are interrelated. Therefore, this study gives additional evidence for planning appropriate intervention in drinking alcohol and khat chewing HIV infected patient who are on ART at the clinic. Participants were enrolled from government ART clinics which might not be representative for patients who do not attend government ART.

The cross-sectional nature of the study design might not show the cause and effect relationships between HIV-related perceived stigma and variables.

## 5 Implications and relevance

The study has tried to determine the prevalence of HIV-related perceived stigma among people living with HIV attending Anti-retroviral clinic is very high. It is very appalling having the prevalence of HIV-related perceived stigma among people living with HIV attending Anti-retroviral clinic who are hypothetical to handover and withstand the countries health development system. Of great concern is the large numbers of patients with living HIV who have HIV-related perceived stigma remain undetected in the study area. Therefore; this calls a holistic approach for the prevention and intervention of HIV-related perceived stigma. Emphasis should also be given for HIV-related perceived stigma.

## 6 Conclusion

The prevalence of HIV-related perceived stigma is high in the study among patients with living HIV. Of great concern is the large numbers of patients with living HIV who have HIV-related perceived stigma remain undetected in the study area.

Being female, Patients who are age groups 25–30 years, age groups 31–39 years, divorced marital status, widowed marital status, Primary educational status and Study participants those who use alcohol were more likely to have HIV-related perceived stigma. Therefore; this calls a holistic approach for the prevention and intervention of HIV-related perceived stigma. Emphasis should also be given for HIV-related perceived stigma.

## Ethical approval and consent to participate

Ethical clearance was obtained from the Institutional Review Board (IRB) of Dilla University. Written consent was obtained from each participant during data collection. All participants were well informed about the aims and purpose of the study, those participants were informed that as the right is given to the study participants to refuse and stop or withdraw from participation at any time during data collection without loss of any entitlement.

## Consent for publication

Not applicable.

## Availability of data and material

The data used to support the findings of this study are included in the article. The data used to support the finding of this study are included within the manuscript can be accessed from the Author “**Yigrem Ali**” upon request through the email address of alyigrem@gmail.com.

## Funding

No funding was obtained from any organization.

## Author contribution

YA&DT conceived the research question, participated in the proposal development, data collection, analysis, interpretation, critically reviewed and approved the manuscript.

## Declaration of competing interest

The author declared that he has no known competing for financial interests or personal relationships that could have appeared to influence the work reported in this paper**.**

## Authors' information (optional)

1*Department of Psychiatry, college of health science and medicine, Dilla University, Dilla, Ethiopia.

## Registration of research studies

This study was registered research registry with the registration number (researchregistry7112).
