# Supplementary material for: HIV-related perceived stigma and associated factors among patients with HIV, Dilla, Ethiopia: A cross-sectional study
Source: Ann Med Surg (Lond). 2021 Oct 8;71:102921. doi: 10.1016/j.amsu.2021.102921 (PMC8515236; doi:10.1016/j.amsu.2021.102921)
Supplement: Multimedia component 1 [file mmc1.docx]

| The STROCSS 2019 Guideline | | |
| --- | --- | --- |
| Item no. | **Item description** | **Page** |
| TITLE | | |
| 1 | HIV-related Perceived Stigma and Associated Factors among Patients with HIV, Dilla, Ethiopia: A Cross-sectional Study | 1 |
| ABSTRACT | | |
| 2a | Introduction: Understanding HIV-related perceived stigma has importance in improving the quality of patients and provides a better tackling of HIV stigma. Therefore; the study aimed to assess the prevalence and associated factors of perceived stigma among Patients with HIV attending the clinic at Dilla University Referral Hospital in Ethiopia 2019. | 2 |
| 2b | Method: In this Institution based cross-sectional study, a 10-item perceived HIV stigma scale was used to assess HIV-related perceived stigma. Oslo social support scale was used to assess social support related factors. Bivariate and multivariate binary logistic analysis was done to identify associated factors to HIV-related perceived stigma. | 2 |
| 2c | Results: The prevalence of HIV-related perceived stigma by using perceived HIV stigma scale among patients with living HIV was 42.7%. Patients who are age groups 25-30 years (AOR=2.8, 95% CI: 5.72-11.5), age groups 31-39 years (AOR=1.11, 95% CI: 1.26,4.65), Females (AOR= 2.4, 95% CI: 1.28 - 4.33), divorced marital status (AOR= 8.9, 95% CI: 3.52-10.61), widowed marital status (AOR= 3.0, 95% CI: 2.74-7.60), Primary educational status (AOR=7.5,95% CI: 3.45-9.74) and Study participants those who use alcohol (AOR=1.0 95% CI: 1.57-2.11) were more likely to have HIV-related perceived stigma. | 2 |
| 2d | Conclusion: This calls a holistic approach to the prevention and intervention of HIV-related perceived stigma. Emphasis should also be given for HIV-related perceived stigma. | 2 |
| INTRODUCTION | | |
| 3 | Introduction: HIV-related perceived stigma my lead to a series of consequences such as non-disclosure of HIV infection seclusion, depressive symptoms, and suicidal ideation and attempt. Due to this effect, PLWH has to cope both with the manifestations of the disease, complex treatment regimen and societal stigma at the same time.  Today, evidence on prevalence and associated factors HIV-related perceived stigma among patients with HIV attending ART clinic is still in demand. Therefore; the study aimed to assess the prevalence and associated factors of perceived stigma among patients with HIV attending the clinic at Dilla University Referral Hospital. | 3 |
| METHODS | | |
| 4a | The study was conducted based on the Strengthening the Reporting of Cohort Studies in Surgery (STROCSS 2019 Guideline) protocols. This study was registered research registry with the registration number (researchregistry7112) | 4 |
| 4b | Ethical approval was obtained from the Institutional Review Board of Dilla University and Referral Hospital. The purpose and importance of the study were explained to each participant before they proceed into actual activities. Confidentiality was maintained by anonymous questionnaire and informed consent was obtained from each participant | 6 |
| 4c | Protocol: The study was conducted based on the Strengthening the Reporting of Cohort Studies in Surgery (STROCSS 2019 Guideline) protocols. This study was registered research registry with the registration number (researchregistry7112) | 4 |
| 4d | Patient Involvement in Research  Patients receiving inpatient treatment and critically ill patients with the difficulty of communication were excluded. | 4 |
| 5a | Study Design: An institutional-based cross-sectional study was conducted at Dilla University Referral Hospital Anti-retroviral clinic from April- May 2019 | 4 |
| 5b | Setting: Dilla University Referral Hospital is found in Dilla Town (the capital of Gedeo Zone) Southern National’s Nationalities and People Region and away 360 km from Addis Ababa, the capital city of Ethiopia. | 4 |
| 5c | Cohort Groups: An institutional-based cross-sectional study was conducted | 4 |
| 5d | Subgroup Analysis: The study was cross-sectional study | 4 |
| 6a | Participants: Patients receiving inpatient treatment and critically ill patients with the difficulty of communication were excluded. | 4 |
| 6b | Recruitment: The study was conducted at Dilla University Referral Hospital Anti-retroviral clinic from April- May 2019 | 4 |
| 6c | Sample Size: The sample size for this study was 403. The study also used a systematic random sampling technique to select study subjects | 5 |
| INTERVENTION AND CONSIDERATIONS | | |
| 7a | Pre-intervention Considerations: The study planned to tackle the perceived related stigma among patients living with HIV. | 3 |
| 7b | Intervention: The study aimed to have holistic approach for prevention and intervention of HIV-related perceived stigma. | 3 |
| 7c | Intra-Intervention Considerations: The study showed the prevalence of perceived stigma among patients living with HIV and were treated at the time study period with mental health professionals. | 6 |
| 7d | Operator Details: The training was given to the data collectors and supervisors on the data collection tool and sampling techniques | 6 |
| 7e | Quality Control: The pretest was done on 5% of the sample size. The training was given to the data collectors and supervisors on the data collection tool and sampling techniques. Supervision was held regularly during the data collection period by the researcher. The data were cross-checked for completeness and consistency daily. | 6 |
| 7f | Post-Intervention Considerations: The study calls a holistic approach for the prevention and intervention of HIV-related perceived stigma. Emphasis should also be given for HIV-related perceived stigma. | 15 |
| 8 | Outcomes: The prevalence of perceived related stigma is the primary outcome of the study. | 5 |
| 9 | Statistics: Descriptive statistics were used to summarize tables and figures and statistical summary measures were used for presentation. Association of HIV-related perceived stigma variables and demographic characteristics were analyzed using chi-square, fisher’s exact test, and binary logistic regression with odds ratio and 95% CI in the univariate analysis. Multivariate logistic regression analysis was carried out to examine the associations between each independent variable and the outcome variable. The model was checked for fitness with R-squared value was an R-squared value greater than 50% considered as good. Hosmer and Lemshow goodness of fit test was also used to check the model fitness. All variables with a p-value of ≤ 0.25 in the bivariable analysis were considered as the candidate for multivariable regression to control possible confounders. Finally, variables with a p-value of <0.05 were as having a statistically significant association with HIV-related perceived stigma at corresponding 95% CI. | 6 |
| RESULTS | | |
| 10a | Participants: A total of 403 participants were interviewed and responded for questionnaires with response rate was 100%. | 7 |
| 10b | Participant Comparison: Most of the study subjects 206 (51.1%) participants were females. 135 (33.5%) respondents were at the age of >39 years, 206 (51.1%) were married and 193 (47.9%) respondents were orthodox in religion. Concerning ethnicity, 193 (47.6%) and 112(27.8%) of them were from Oromo and Gedeo ethnic group, respectively. The majority 182 (45.2%) respondents had secondary school education, 124 (30.8%) participants were a government employee. Majority of the total respondents 161 (40.0%) of them were living with their children 159 (39.5%) were have poor social support and 244 (60.5%) were have strong social support more than half of the respondents 223 (55.3%) were use substance and 192 (47.6%) were the second stage of HIV | 7 |
| 10c | Intervention: The overall prevalence of perceived stigma was found very high. this calls a holistic approach for the prevention and intervention of HIV-related perceived stigma. Emphasis should also be given for HIV-related perceived stigma. | 15 |
| 11a | Outcomes: The overall prevalence of Perceived Stigma was found to be 42.7%. | 10 |
| 11b | Tolerance: the study was cross-sectional design study | 4 |
| 11c | Complications: medication non-adherence is the result of perceived stigma among patients living with HIV. | 11 |
| 12 | Key Results: After adjusting for possible covariates, age, sex, marital status, ethnicity, educational status, occupational, living status, HIV stage was significantly associated with HIV-related perceived stigma among patients living with HIV with p-value<0.05. | 11 |
| DISCUSSION | | |
| 13 | Discussion: The study has tried to determine the prevalence of HIV-related perceived stigma and associated factors among people living with HIV attending Anti-retroviral clinic at Dilla University Referral Hospital .thus the prevalence of HIV-related perceived stigma was found to be 42.7%. | 13 |
| 14 | Strengths and Limitations: The cross-sectional nature of the study design might not show the cause and effect relationships between HIV-related perceived stigma and variables | 14 |
| 15 | Implications and Relevance: The study has tried to determine the prevalence of HIV-related perceived stigma among people living with HIV attending Anti-retroviral clinic is very high. It is very appalling having the prevalence of HIV-related perceived stigma among people living with HIV attending Anti-retroviral clinic who are hypothetical to handover and withstand the countries health development system. Of great concern is the large numbers of patients with living HIV who have HIV-related perceived stigma remain undetected in the study area. Therefore; this calls a holistic approach for the prevention and intervention of HIV-related perceived stigma. Emphasis should also be given for HIV-related perceived stigma | 14 |
| CONCLUSION | | |
| 16 | Conclusions: The prevalence of HIV-related perceived stigma is high in the study among patients with living HIV. Of great concern is the large numbers of patients with living HIV who have HIV-related perceived stigma remain undetected in the study area.  Being female, Patients who are age groups 25-30 years, age groups 31-39 years, divorced marital status, widowed marital status, Primary educational status and Study participants those who use alcohol were more likely to have HIV-related perceived stigma. Therefore; this calls a holistic approach for the prevention and intervention of HIV-related perceived stigma. Emphasis should also be given for HIV-related perceived stigma. | 15 |
| DECLARATIONS | | |
| 17a | Conflicts of interest  The author declared that he has no known competing for financial interests or personal relationships that could have appeared to influence the work reported in this paper. | 16 |
| 17b | Funding  No funding was obtained from any organization | 16 |
